# Characterization of a humanized mouse model of Charcot-Marie-Tooth type 1A for the discovery of human *PMP22*-targeting drugs

**DOI:** 10.3389/fneur.2025.1658204

**Published:** 2025-10-29

**Authors:** Atsuki Taruta, Shin-ichi Matsumoto, Yasushi Masuda, Tetsuaki Hiyoshi, Akina Harada, Yohei Kosugi, Masato Nakashima

**Affiliations:** 1 Neuroscience Drug Discovery Unit, Takeda Pharmaceutical Company Limited, Fujisawa, Japan; 2 Preclinical and Translational Sciences, Takeda Pharmaceutical Company Limited, Fujisawa, Japan

**Keywords:** Charcot–Marie-Tooth type 1A, *PMP22*, humanized model, demyelination, electrophysiology, muscle deterioration

## Abstract

**Background and aims:**

Charcot–Marie-Tooth type 1A (CMT1A) is the most common inherited demyelinating peripheral neuropathy caused by duplication of the peripheral myelin protein 22 (*PMP22*) gene. Although there is currently no approved treatment for CMT1A, reducing PMP22 expression has emerged as a promising therapeutic approach. The PMP22-C3 mouse model is a widely used CMT1A model that carries human *PMP22* (*hPMP22*) and mouse *Pmp22* (*mPmp22*) genes, complicating the relationship between reduced PMP22 levels and the recovery of phenotypes by drug candidates targeting only human PMP22. To address this, we developed humanized C3 mouse lines lacking the *mPmp22* gene. Here, we characterized these models to confirm their utility as novel disease models for CMT1A.

**Methods:**

Heterozygous (hetero-humanized) and homozygous (homo-humanized) *hPMP22* transgenic mice, with an *mPmp22* homozygous knockout background, were investigated using biochemical, electrophysiological, histopathological, and behavioral analyses.

**Results:**

Homo-humanized mice exhibited abnormal mRNA expression of myelin-related genes, slow nerve conduction velocity, reduced compound muscle action potential, demyelinated peripheral nerves, higher levels of plasma neurofilament light chain, muscle weakness and motor/balance disabilities, alterations in electrical impedance myography, and muscle fiber atrophy. In contrast, the hetero-humanized mice did not display any of the previously described impairments.

**Interpretation:**

Homo-humanized mice reflect various aspects of CMT1A characteristics in an *hPMP22* gene dosage-dependent manner. This model will help us better understand the relationship between *PMP22* reduction levels and the recovery of CMT1A-related phenotypes, contributing to the translation of preclinical findings into clinically relevant human treatments and dosing strategies.

## 1 Introduction

Charcot–Marie-Tooth type 1A (CMT1A) is the most common autosomal dominant inherited demyelinating peripheral neuropathy, accounting for more than 50% of all CMT cases (1–3). The primary symptoms include distal muscle weakness/atrophy, balance problems, high-arched feet, tremors, and pain (1, 3, 4). CMT1A is caused by a duplication on chromosome 17p11.2, containing the gene encoding peripheral myelin protein 22 kDa (PMP22), which is a critical component of the myelin sheath (4, 5). Proper expression of PMP22 and other myelin-related proteins, such as sterol-C5-desaturase (SC5D), myelin protein zero (MPZ), and POU Class 3 homeobox 1 (POU3F1), is important for the development and maintenance of myelin structure in peripheral nerves (2, 6–8). In patients with CMT1A, increased levels of *PMP22* (1.5–2-fold) and demyelination in peripheral nerves have been demonstrated (9–13). These changes result in slow nerve conduction velocity (NCV) (4, 9, 14, 15) and reduced compound muscle action potential (CMAP) amplitude that reflects axonal dysfunction secondary to demyelination (3, 16). In contrast, heterozygous deletion of *PMP22* (approximately 0.5-fold reduction in expression) leads to another demyelinating neuropathy, Hereditary Neuropathy with Liability to Pressure Palsies (HNPP) (4, 10, 17, 18). Therefore, maintaining PMP22 expression within the appropriate range is essential for the treatment of CMT1A.

Since CMT1A is caused by overexpression of PMP22, directly reducing PMP22 expression, for example, by antisense oligonucleotides (ASO), is expected to be a promising therapeutic approach. Treatment with *PMP22*-targeting ASO or microRNA ameliorated the deficits in CMT1A mouse models, including declines in NCV and CMAP, demyelination in peripheral nerves, and motor disabilities (19, 20); however, an excessive reduction in *PMP22* is associated with a risk of HNPP-like symptoms. Thus, a better understanding of the relationship between PMP22 expression and pathological phenotypes in animal models is important for predicting the therapeutic effects of PMP22-targeting drug candidates.

Transgenic mouse models of CMT1A overexpressing the human *PMP22* (*hPMP22*) gene, including PMP22-C3 mice that contain 3–4 copies of *hPMP22*, have been widely used to elucidate disease mechanisms and facilitate drug discovery (21–23). These models carry the *hPMP22* gene and mouse *Pmp22* (*mPmp22*) gene, which makes it difficult to estimate the level of “total” PMP22 reduction by drug candidates targeting only hPMP22. For instance, the complete elimination of hPMP22 means just “normalization” in PMP22-C3 mice due to the remaining intrinsic mPmp22. In other words, PMP22-C3 mice can lead to a misunderstanding of the functional efficacy and adverse effects of human PMP22-targeting drugs on hPMP22-reducing effects. To address this issue, we generated two humanized PMP22-C3 mouse models that completely lack *mPmp22* gene: heterozygous or homozygous *hPMP22* transgene expression (hetero-humanized with 3–4 copies or homo-humanized with 6–8 copies) in an *mPmp22* homozygous knockout (KO) background. We characterized these mouse lines by investigating peripheral nerve deterioration and subsequent muscle impairment to assess their utility as new CMT1A disease models for a more precise preclinical evaluation of drug candidates specifically targeting hPMP22.

## 2 Materials and methods

### 2.1 Animals

**Figure 1:**
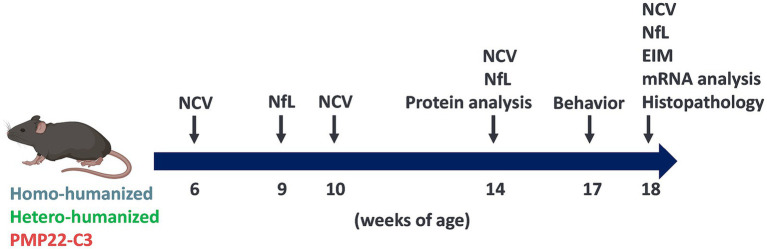
Experimental schedule for characterization of humanized mice. NCV and CMAP assessments were conducted at 6, 10, 14, and 18 weeks of age. Plasma NfL levels were measured at 9, 14, and 18 weeks of age. PMP22 protein levels were investigated at 14 weeks. Analyses of mRNA levels of *PMP22* and myelin-related genes utilized samples from 18-week-old mice. Behavioral testing and EIM measurements were performed at 17 and 18 weeks of age, respectively. Histopathological examinations of the sciatic nerves and GC muscles were conducted at 18 weeks of age.

PMP22-C3 Tg (hetero) mice [B6.Cg-Tg(PMP22)C3Fbas/J] were purchased from Jackson Laboratory (Bar Harbor, ME, USA) with permission for breeding and genetical modifying. They were bred in Axcelead Drug Discovery Partners, Inc. (ADDP, Kanagawa, Japan) and male PMP22-C3 mice and age-matched wild-type (WT) littermates were used. Humanized mice [hPMP22-C3 Tg/mPmp22 KO (ho/ho); homo-humanized, hPMP22-C3 Tg/mPmp22 KO (he/ho); hetero-humanized] were generated by crossing the PMP22-C3 mouse line with *mPmp22* KO mouse line that lacks the full length (31 kbp) *mPmp22* gene using the CRISPR/Cas9 method and supplied by ADDP. In detail, hPMP22-C3 Tg /mPmp22 KO (he/he) mice were initially generated by crossing hPMP22-C3 Tg (hetero) mice with mPmp22 KO (homo) mice using *In Vitro* Fertilization method. Then hPMP22-C3 Tg /mPmp22 KO (he/he) mice were crossed with hPMP22-C3 Tg /mPmp22 KO (wt/he) mice to generate hPMP22-C3 Tg /mPmp22 KO (he/ho) mice. Consequently, hPMP22-C3 Tg /mPmp22 KO (he/ho) mice were bred to generate hPMP22-C3 Tg /mPmp22 KO (ho/ho) mice. The ratio of these mouse lines was broadly consistent with Mendel’s Laws. Genotyping analysis using PCR method (50 °C for 2 min, then 95 °C for 20 s, following 40 cycles of 95 °C, 1 s and 60 °C, 20 s) was conducted to identify the genotypes of each mouse. The following primer sets were used: *mPmp22* (Forward: 5′-GCGTCCTCTTTCATGAATCAAAA, Reverse: 5′-TCTACCCCTAACCTCTGGTTCCT, Probe: 5′-CATTCACGAATTGCC; Thermo Fisher Scientific, Waltham, MA, USA), *hPMP22* (Forward: 5′-GTGCTGCGGCCATCTACAC, Reverse: 5′-GTAGGCGAAACCGTAGGAGTAATC, Probe: 5′-CCGGAGTGGCATCT; Thermo Fisher Scientific). The phenotypic characteristics of these two humanized mice were stable across generations. As the control group for humanized models, age-matched C57BL/6 J Jcl mice (WT), the background mouse line of PMP22-C3 mice and *mPmp22* KO mice were purchased from CLEA Japan, Inc. (Tokyo, Japan). The mice were housed in groups with unrestricted access to food and water and maintained under a 12-h light/dark cycle (lights on at 7:00 a.m.) at a temperature of 22 ± 1 °C. To study the natural history of NCV and CMAP, mice were subjected to continuous NCV and CMAP recording at 6, 10, 14, and 18 weeks of age from the same individuals (*n* = 8–10/group). The mice were then sacrificed, and histopathological analysis and evaluation of mRNA expression were performed. Behavioral assessments and electrical impedance myography (EIM) measurements were performed at 17 and 18 weeks of age, respectively (*n* = 8–10/group). Another group of 14-week-old humanized and PMP22-C3 mice was used for the PMP22 protein assay (*n* = 4/group). The overall experimental schedule is shown in Figure 1. All experimental procedures in this study were conducted in accordance with animal care protocols approved by the Institutional Animal Care and Use Committee of Shonan Health Innovation Park (protocol number: AU-00031034 and AU-00031278), in compliance with *Act on Welfare and Management of Animals* (Act No. 105 of October 1, 1973), harmonized with the American Association for Accreditation of Laboratory Animal Care (AAALAC) International.

### 2.2 Messenger RNA extraction and real-time quantitative polymerase chain reaction (RT-qPCR)

Mice were anesthetized via intraperitoneal injection of a combination of anesthetics (medetomidine, 0.3 mg/kg; midazolam, 4 mg/kg; and butorphanol, 5 mg/kg) and then euthanized by exsanguination. The left sciatic nerve was removed and homogenized using a FastPrep-24 5G homogenizer (MP Biochemicals LLC, Irvine, CA, USA) in Lysing Matrix I tubes (6918–100, MP Biochemicals LLC) filled with QIAzol Lysis Reagent (79,306, Qiagen, Hilden, Germany). Total RNA was extracted using the RNeasy Plus Universal Mini Kit (73,404, Qiagen) according to the manufacturer’s instructions. Total RNA (0.1 μg) was used for complementary DNA synthesis using SuperScript IV VILO Master Mix (11,756,500, Thermo Fisher Scientific). RT-qPCR was conducted using QuantStudio (Thermo Fisher Scientific) with TaqPath qPCR Master Mix, CG (A15297; Thermo Fisher Scientific). Relative copy numbers of *mPmp22* and *hPMP22* were calculated. The mRNA expression levels of myelin-related genes were normalized to those of *Gldn*, a Schwann cell-specific gene, and compared with each WT group. The following primer sets were used: *mPmp22* (Forward: 5′-CTTCCAAATCCTTGCTGGTCTG, Reverse: 5′-GGATGTAGGCGAAGCCATAGG, Probe: 5′-ATGCCACTCACTGTGCCTCACTGTGTAGAT; Sigma-Aldrich, Saint Louis, USA), *hPMP22* (Forward: 5′-CTTGCTGGTCTGTGCGTGAT, Reverse: 5′-ACCGTAGGAGTAATCCGAGTTGAG, Probe: 5′-CATCTACACGGTGAGGCACCCGG; Sigma-Aldrich), *Sc5d* (Mm. PT.58.33540072; IDT, San Jose, CA, USA), *Mpz* (Mm. PT.58.5771188; IDT), *Pou3f1* (Mm. PT.58.33607006. g; IDT), *Gldn* (Mm. PT.58.43925599; IDT).

### 2.3 Quantitative evaluation of mPmp22 and hPMP22 protein expression

After euthanasia by exsanguination, the left sciatic nerve was removed. 2.5% homogenate of sciatic nerve was homogenized by Precellys Evoluation (Beritin, France) with lysis buffer including 50 mM Tris pH 7.5, 4% sodium dodecyl sulfate, 150 mM NaCl, Complete (04693132001, Roche, Switzerland), and PhosSTOP (04906837001, Roche). After incubation at room temperature (20–25 °C) for 30 min, the samples were subjected to ultrasonication at 40 kHz for 10 min and then incubated at 95 °C for 10 min using an Eppendorf ThermoMixer C (Eppendorf, Germany). After cooling to room temperature, the samples were centrifuged at 21,600 × g for 10 min at room temperature. The protein concentration in the supernatant was measured using a bicinchoninic acid assay. Sciatic nerve (7.5 μg) was mixed with 1 M phosphoric acid buffer (10 μL), 5 M NaCl (10 μL), and cold acetone (0.5 mL) at −30 °C for 2 h. After centrifugation (21,600 × g, 4 °C, 20 min), the supernatant was completely removed and the precipitant was kept at room temperature for 15 min. The precipitant was dissolved in 8 M urea (50 μL), 0.5 M Tris(2-carboxyethyl) phosphine (5 μL), and 0.5 M iodoacetamide (5 μL) and incubated for 15 min at room temperature. This was followed by sonication for 10 min and a subsequent incubation for 10 min at 37 °C. Next, 800 μL of 0.1 M Tris–HCl (pH 8.0) was added, and proteolytic digestion was performed using 12.5 μg of chymotrypsin (Promega, WI, USA) overnight at 25 °C. The resulting sample was mixed with 4% phosphoric acid (200 μL) and 100 mg/mL of internal standard peptides (10 μL), then purified via solid-phase extraction using Oasis MCX cartridges (Waters, MA, USA). The final eluted solution was dried by nitrogen gas. The residue was reconstituted in 200 μL of a formic acid/acetonitrile/purified water (1,40,960, v/v/v), and an aliquot of 0.2 μL was injected into an EASY-nLC 1,200 system/Q Exactive HF-X Orbitrap mass spectrometer (Thermo Fisher Scientific). The signature peptides were monitored in parallel reaction monitoring mode. The detailed analytical conditions are listed in Supplementary Tables S1, S2.

### 2.4 NCV and CMAP measurements

NCV and CMAP were recorded from the right sciatic nerve of WT and humanized mice using a Natus UltraPro S100 (Natus Medical Inc., Middleton, WI, USA). Disposable monopolar needle electrodes (TECA^®^ Elite, 902-DMF25-TP (28G), Natus Medical Inc.) were used for stimulating and recording. Mice were anesthetized with 1.5–2.0% isoflurane on a heating pad to maintain body temperature. To record the NCV and CMAP, a sensing recording electrode was inserted into the tibialis anterior muscle, and the reference electrode was inserted into the ipsilateral plantar muscle. For stimulation, the stimulating cathodes were subcutaneously placed at the sciatic nerve, 4 mm (distal) and 16 mm (proximal) from the sensing recording electrode. The anode was inserted into the contralateral plantar muscle. Square-wave pulses of 0.1 ms duration were delivered to the distal and proximal sites at a 0.5 Hz frequency, and each CMAP was measured at supramaximal stimulation. The NCV was calculated from the difference in peak latencies between the distal and proximal CMAP responses and the distance (12 mm) between the stimulating electrodes. The CMAP evoked by proximal stimulation was used to analyze the CMAP amplitude.

### 2.5 Sciatic nerve histopathology

The right sciatic nerve was immersed in 4% paraformaldehyde phosphate buffer solution (163–20,145, FUJIFILM Wako Pure Chemical Corp., Osaka, Japan) for 5–7 days at 4 °C. The fixative was replaced with 0.01 mol/L phosphate-buffered saline and stored at 4 °C until the histopathological analysis. For morphometric assessment of axons and myelin, the sciatic nerve was embedded in resin and sectioned transversely (0.5 μm thick), and stained with toluidine blue (104,172, Merck, Darmstadt, Germany). Digital images were obtained using a digital slide scanner (NanoZoomer S60; Hamamatsu Photonics, Shizuoka, Japan), and the inner and outer areas/diameters of the axon and myelin were subsequently analyzed using HALO image analysis software (Indica Labs, Albuquerque, NM, USA). The g-ratio of each axon was calculated as a ratio between axon outer diameter and inner diameter.

### 2.6 Measurement of plasma NfL levels

Blood samples were collected from the jugular vein of the mouse, which was gently restrained by hand, at 9 and 14 weeks of age, and from the abdominal vena cava of the mouse anesthetized with the combination anesthetic described above at 18 weeks of age. Approximately 200 μL of whole blood was collected using a 1 mL syringe with a 27-guage needle coated with 0.1 mol/L EDTA-2Na (344–09255, Dojindo Laboratories, Kumamoto, Japan) and kept on ice. After the centrifugation at 17,400 × g at 4 °C for 1 min, 40 μL of plasma was collected into a Protein LoBind^®^ Tube (0030108434, Eppendorf), then chilled on dry ice and stored at −80 °C until analysis. NfL levels in plasma were measured using an HD-X analyzer (Quanterix Corporation, Massachusetts, USA) with a Simoa NF-Light™ V2 Advantage Kit (104,073, Quanterix) according to the manufacturer’s instructions.

### 2.7 Behavioral assessments

#### 2.7.1 Grip strength test

The grip strengths of the forelimbs and hindlimbs were measured using a grip strength meter (MK-380CM; Muromachi Kikai Co., Ltd., Tokyo, Japan). The apparatus consisted of a triangular metal bar or a T-shaped metal bar connected to a strength transducer to measure the grip strength of the forelimbs or hindlimbs, respectively. Each mouse was held by its tail and forced to grasp the metal bar with its forelimbs or hindlimbs. The mouse was then gently pulled backward with a consistent force until the grip was lost. This step was repeated five times, and the data were averaged after excluding the maximal and minimal values.

#### 2.7.2 Beam walking test

The beam walking test was performed to evaluate gait and balance. A square metal bar (width 1.2 cm, length 85 cm) with a dark box at the arrival point was placed 60 cm above the ground. Each mouse was habituated to the box for 5 min and then trained to walk to the box five times. The test session was performed once, at least 1 h later. The mice were placed at the departure point and allowed to walk to the box. The transit time and the number of slips were measured. Trials in which the mouse fell off, paused (over 5 s), or ran backward were excluded, and the mouse was retested.

#### 2.7.3 Rotarod test

To evaluate motor coordination, the mice were tested on a rotarod apparatus (MK-670; Muromachi Kikai Co., Ltd., Tokyo, Japan). Each mouse was trained on a rotating rod (three 5-min sessions at 4, 8 and 15 rpm). During the test trials (at least 1 h later), each mouse was placed on the rotarod at increasing speeds from 4 to 40 rpm for 300 s. The test trials were performed three times, at least 15 min apart. The latency to fall off the rotarod during Trials 2 and 3 was recorded, and the mean value was used in the analysis.

### 2.8 EIM measurement

EIM measurements were performed as previously described (24). Briefly, mice were anesthetized with 1.5–2.0% isoflurane and placed on a heating pad to maintain their body temperature. The left hindlimb was shaved and pretreated with skin preparation gel (Nuprep^®^; Weaver and Company, Aurora, CO, USA) to reduce impedance, and positioned at a 45° angle. After application of an electrode cream (SignaCreme^®^; Parker Labs, Fairfield, NJ, USA), a four-electrode array with a weight of 20 g was placed over the gastrocnemius (GC) muscle in the longitudinal direction and then, a weak electrical current with multi-frequencies was applied. The EIM parameters (reactance, resistance, and phase) were obtained using an impedance spectroscopy system (mScan-V^™^; Myolex Inc., Boston, MA, USA). Measurements were performed three times and averaged within the 1–10,000 kHz range. Values from 10 to 1,000 kHz were used for the analysis to avoid artifacts.

### 2.9 Skeletal muscle histopathology

The hindlimbs were removed from each mouse and immersed in 4% paraformaldehyde phosphate buffer solution (163–20,145; FUJIFILM Wako Pure Chemical Corp., Osaka, Japan). After 24 h, the fixative was replaced with 0.01 mol/L phosphate-buffered saline and stored at 4 °C. To assess muscle pathology, GC muscle tissues were assessed using laminin immunohistochemical staining as previously described (24). Digital images were obtained using a digital slide scanner (NanoZoomer S60; Hamamatsu Photonics, Shizuoka, Japan), and the cross-sectional area (CSA) and muscle fiber count were subsequently analyzed using HALO image analysis software (Indica Labs).

### 2.10 Data analysis

Statistical analyses were performed using the SAS software (version 9.4; SAS Institute Japan, Tokyo, Japan). For multiple comparison for *PMP22* mRNA and protein levels, one-way ANOVA with a *post hoc* Bonferroni’s test was utilized. To compare two groups genetic differences between humanized or PMP22-C3 and WT mice, data were assessed using Student’s t-test for homogeneous data or Mann–Whitney U test for nonhomogeneous data. For the analysis of repeated measures data of NAV, CMAP, and plasma NfL, two-way Repeated Measures ANOVA with a *post hoc* Bonferroni’s test was performed. Statistical significance was set at *p* < 0.05. Data were reported as the mean ± standard error of the mean (SEM).

## 3 Results

### 3.1 Expression of *mPmp22* or *hPMP22* mRNA and protein in sciatic nerves of humanized mice and PMP22-C3 mice

**Figure 2:**
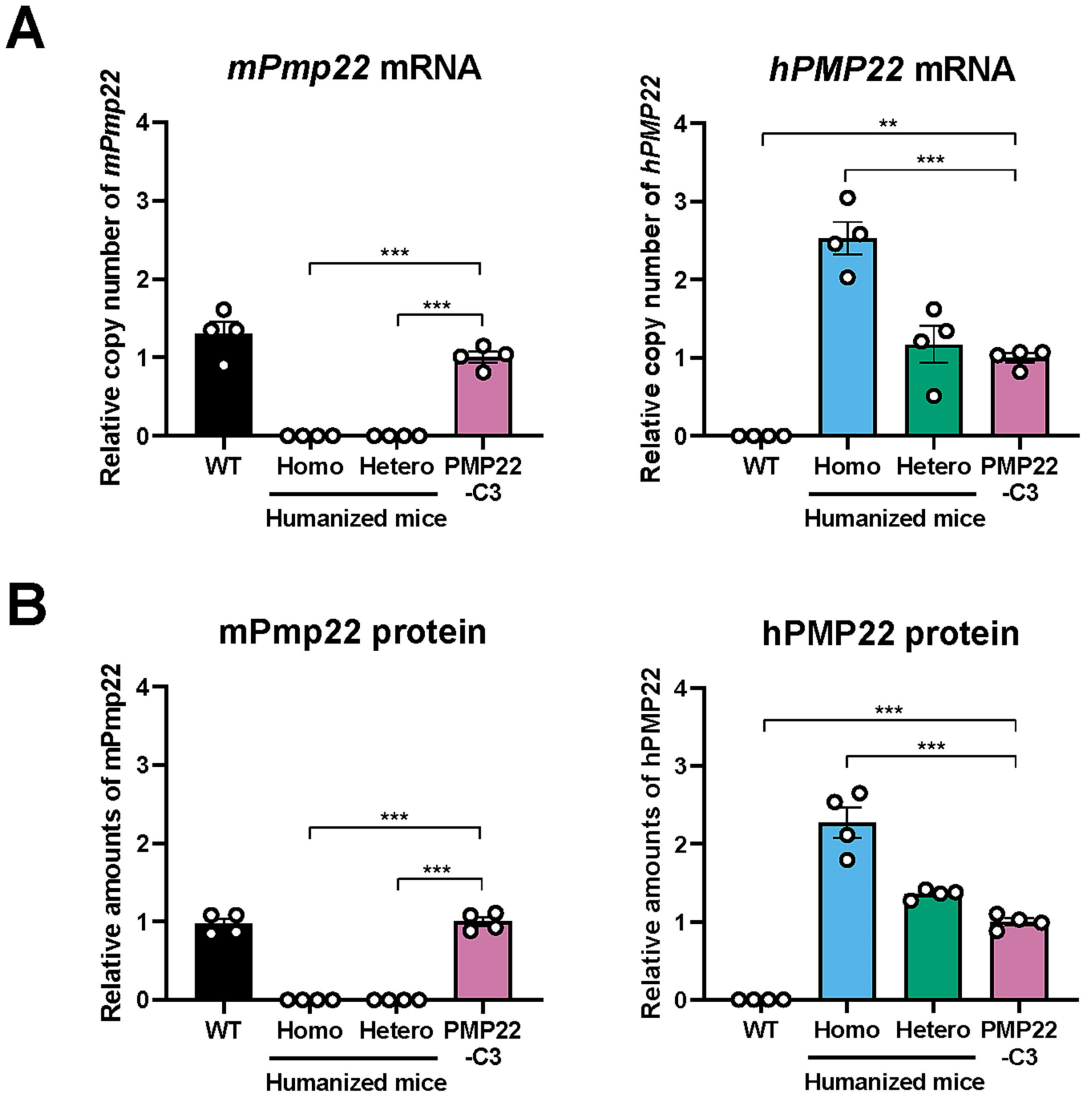
Expression of mouse *Pmp22* and human *PMP22* mRNA and protein in the sciatic nerve of WT, humanized, and PMP22-C3 mice. **(A)** Relative copy numbers of mouse *Pmp22* and human *PMP22* mRNA or **(B)** relative amounts of mouse Pmp22 and human PMP22 protein were analyzed in the sciatic nerve of WT (C57BL/6 J), homo-humanized, hetero-humanized, and PMP22-C3 mice (*n* = 4/group) at 18 or 14 weeks of age, respectively. Data are shown as the ratio to PMP22-C3 mice and as mean ± SEM. Statistical comparisons were performed using one-way ANOVA with a *post hoc* Bonferroni’s test (^**^p < 0.01, ^***^p < 0.001 vs. PMP22-C3 mice). WT, wild-type; SEM, standard error of the mean.

To confirm the genetic characteristics of humanized mice, we investigated the mRNA expression of *mPmp22* and *hPMP22* in the sciatic nerves of WT, humanized, and PMP22-C3 mice at 18 weeks of age using RT-qPCR. *mPmp22* was not detected in hetero-humanized or homo-humanized mice, whereas WT and PMP22-C3 mice showed similar levels of intrinsic *mPmp22* expression (Figure 2A). The expression level of *hPMP22* in hetero-humanized mice was similar to that in PMP22-C3 mice. In contrast, homo-humanized mice showed higher levels of *hPMP22* expression than PMP22-C3 or hetero-humanized mice (2.5-fold increase compared to PMP22-C3 mice). Consistent with the mRNA expression of *mPmp22* and *hPMP22*, hetero- and homo-humanized mice showed no expression of mPmp22 protein, and homo-humanized mice exhibited a higher level of hPMP22 protein than PMP22-C3 mice or hetero-humanized mice (2.3-fold increase compared to PMP22-C3 mice) (Figure 2B). These data confirmed that humanized mice had no *mPmp22* gene or protein and that gene dosage-dependent *hPMP22* mRNA and protein expression was observed in the sciatic nerve.

### 3.2 Alterations in transcript levels of myelin-related genes in humanized mice

**Figure 3:**
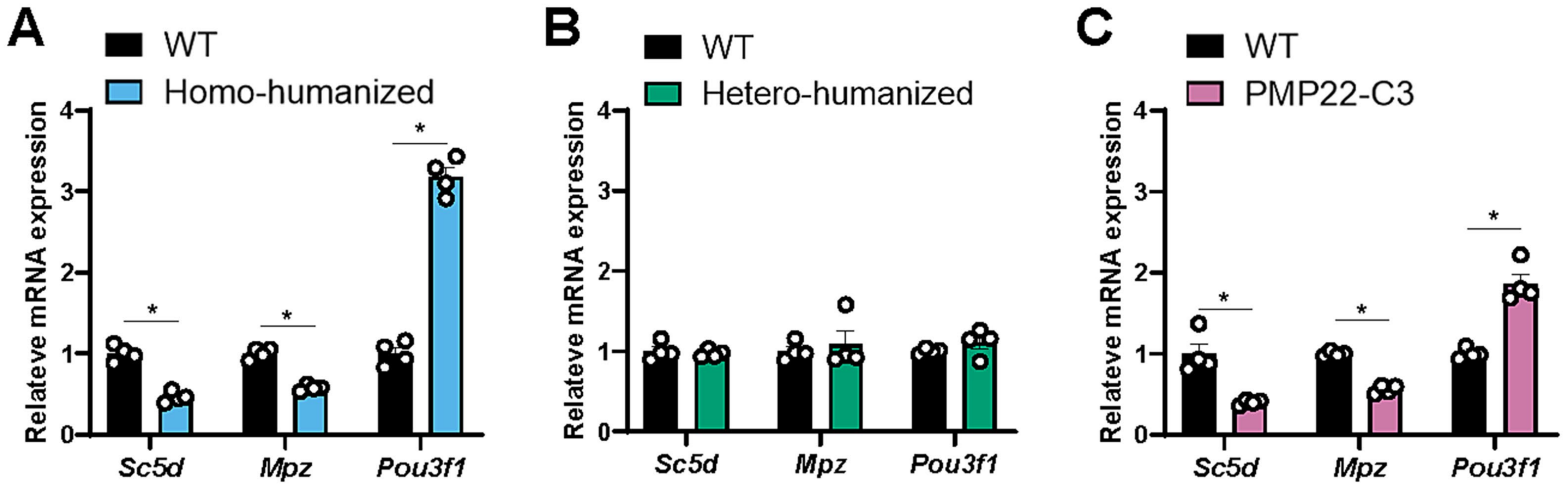
Quantitative analysis of myelin-related genes in the sciatic nerve of humanized mice and PMP22-C3 mice. Transcript levels of myelin-related genes (*Sc5d*, *Mpz*, and *Pou3f1*) were analyzed in the sciatic nerves of WT, **(A)** homo-humanized, **(B)** hetero-humanized, and **(C)** PMP22-C3 mice at 18 weeks of age using RT-qPCR. Data are shown as the ratio to the WT group and as mean ± SEM (*n* = 4/group). Statistical comparisons were performed using Mann–Whitney U test (^*^p < 0.05 vs. WT mice). RT-qPCR, real-time quantitative polymerase chain reaction; WT, wild-type; SEM, standard error of the mean.

Transcriptional changes in several myelin-related genes, such as *Sc5d*, *Mpz*, and *Pou3f1*, were investigated in the sciatic nerves of humanized and PMP22-C3 mice because changes in the transcript levels of these genes have been reported in the CMT1A rodent model (19). The mRNA expression of these genes in the sciatic nerve was assessed using RT-qPCR at 18 weeks of age. In homo-humanized mice, the transcript levels of *Sc5d* and *Mpz* were decreased, and *Pou3f1* was increased relative to those in WT mice (*Sc5d*: 0.46-fold reduction, *Mpz*: 0.58-fold reduction, *Pou3f1*: 3.2-fold increase; *p* < 0.005) (Figure 3A), consistent with those in PMP22-C3 mice (Figure 3C) and a previous report (19). In contrast, hetero-humanized mice showed transcript levels of these genes comparable to those in WT mice (Figure 3B). These data suggest that the mRNA expression levels of myelin-related genes changed depending on *PMP22* levels in the sciatic nerve of humanized mice.

### 3.3 Longitudinal deficits in NCV and CMAP in humanized mice

**Figure 4:**
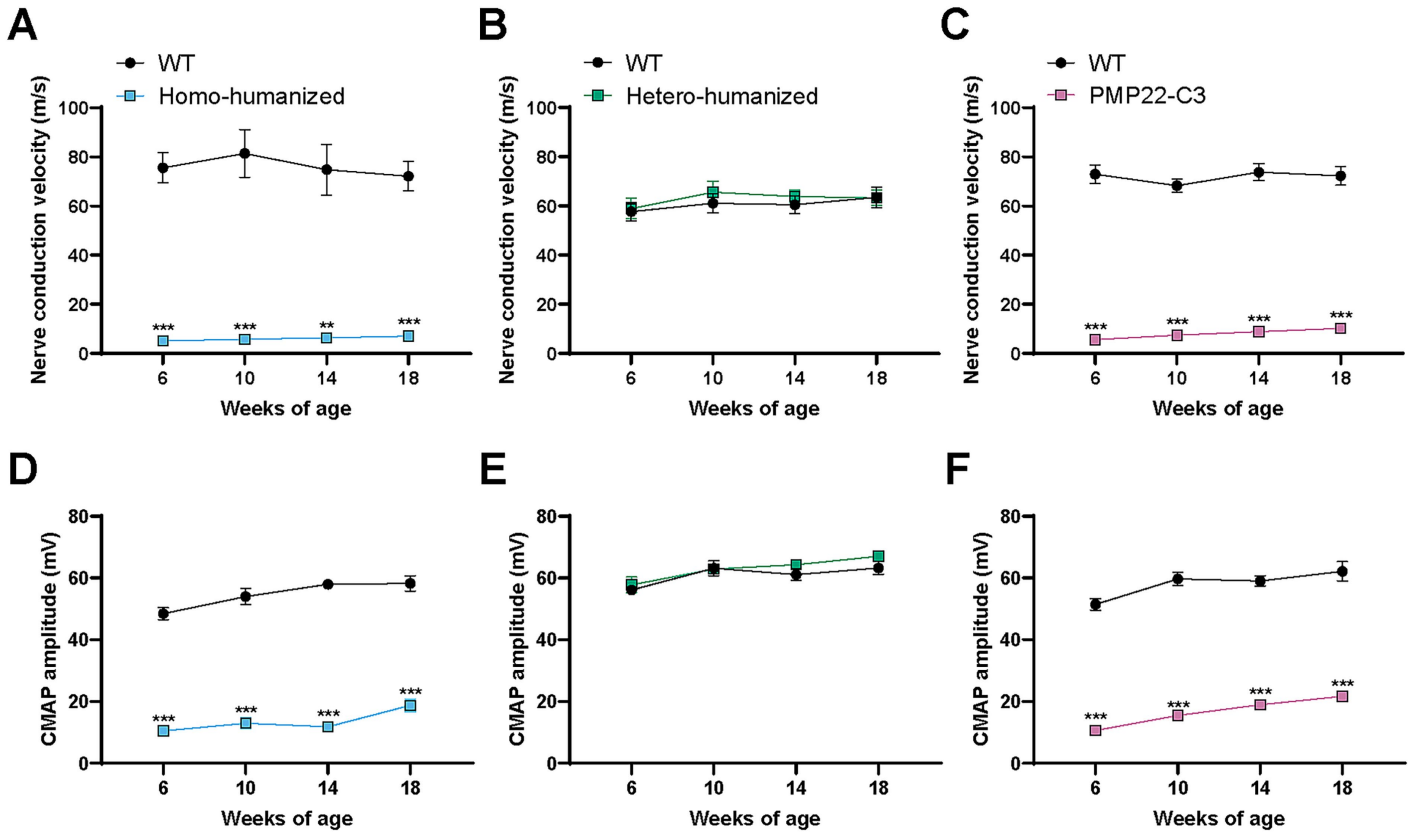
Longitudinal data of NCV and CMAP in humanized mice and PMP22-C3 mice. NCV and CMAP were measured repeatedly at 6, 10, 14, and 18 weeks of age in the WT, **(A,D)** homo-humanized, **(B,E)** hetero-humanized, and **(C,F)** PMP22-C3 mice. Data are represented as mean ± SEM (*n* = 8–10/group). Statistical comparisons were performed using two-way Repeated Measures ANOVA with a post hoc Bonferroni’s test (^**^p < 0.01, ^***^p < 0.001 vs. WT mice). NCV, nerve conduction velocity; CMAP, compound muscle action potential; WT, wild-type; SEM, standard error of the mean.

Patients with CMT1A and animal models of this disease exhibit electrophysiological impairment of peripheral nerves due to demyelination (9, 15, 21, 22). To investigate functional changes in the peripheral nerves, NCV and CMAP were repeatedly measured in humanized and PMP22-C3 mice at 6, 10, 14, and 18 weeks of age. Individual values of NCV and CMAP are shown in Supplementary Table S3. Robustly slower NCV (6.8, 7.1, 8.5, and 9.7% of WT mice at 6, 10, 14, and 18 weeks of age, respectively; *p* < 0.01 or 0.001) and reduced CMAP amplitude (21.7, 24.0, 20.4, and 32.3% of WT mice at 6, 10, 14, and 18 weeks of age, respectively; *p* < 0.001) were observed in homo-humanized mice compared to WT mice (Figures 4A,D), reflecting demyelination in the sciatic nerve and indicating an abnormality in the lower motor unit of this model. These results were comparable to those observed in the PMP22-C3 mice (Figures 4C,F). In contrast, hetero-humanized mice showed no differences from WT mice in NCV (102.2, 107.4, 105.5, and 99.7% of WT mice at 6, 10, 14, and 18 weeks of age, respectively) or CMAP (103.0, 99.6, 105.2, and 106.0% of WT mice at 6, 10, 14, and 18 weeks of age, respectively) at all ages (Figures 4B,E). These findings suggest that homo-humanized mice show peripheral nerve dysfunction similar to that in PMP22-C3 mice, which depends on the total amount of *PMP22* expression.

### 3.4 Morphological changes in the sciatic nerve of humanized mice

**Figure 5:**
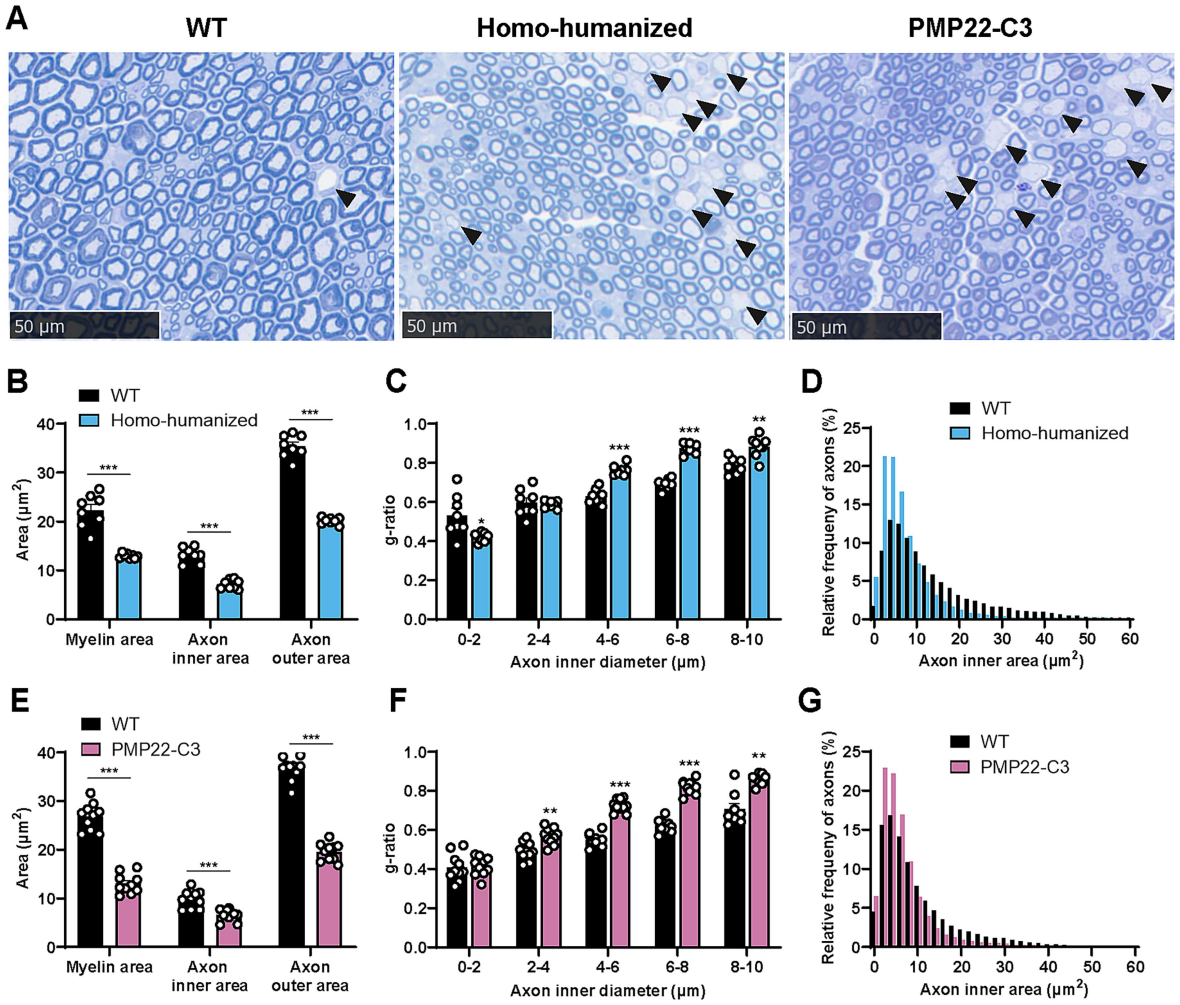
Histopathological analysis in the sciatic nerve of homo-humanized mice and PMP22-C3 mice. **(A)** Representative images of toluidine blue-stained sciatic nerve in WT, homo-humanized, and PMP22-C3 mice at 18 weeks of age. Demyelinated axons are pointed with black arrowheads. **(B,E)** Myelin area, axon inner area, and outer area in WT and **(B)** homo-humanized mice or **(E)** PMP22-C3 mice. **(C,F)** The g-ratio of each axon in WT and **(C)** homo-humanized mice or **(F)** PMP22-C3 mice was analyzed by axon size. **(D,G)** Comparison of histograms for axon inner area in the sciatic nerve of WT and **(D)** homo-humanized mice or **(G)** PMP22-C3 mice. Analyzed axon frequencies from all animals are plotted for each given axon inner diameter with a 2 μm^2^-bin. Data are represented as mean ± SEM (*n* = 8–10/group). Statistical comparisons were performed using Mann–Whitney U test (^*^p < 0.05, ^**^p < 0.01, ^***^p < 0.001 vs. WT mice). WT, wild-type; SEM, standard error of the mean.

As homo-humanized and PMP22-C3 mice showed changes in myelin-related genes and demyelination-related nerve dysfunction in the sciatic nerve, histopathological analyses of the sciatic nerve were performed using these mice at 18 weeks of age. In homo-humanized mice, many demyelinated axons were observed (Figure 5A, middle panel, arrowhead), and quantitative analysis revealed a smaller myelin area (58.3% of WT mice, *p* < 0.001) than in WT mice (Figure 5B). To further investigate the characteristics of demyelination in homo-humanized mice, the g-ratio of each axon was calculated and analyzed by axon size (Figure 5C). Homo-humanized mice exhibited higher g-ratio, especially in larger axons, consistent with previous reports for PMP22-C3 mice (25, 26). In addition, the axonal inner (54.4% of WT mice, *p* < 0.001) and outer areas (56.8% of WT mice, *p* < 0.001) were significantly smaller in homo-humanized mice than in WT mice (Figure 5B), and the distribution of axon size in homo-humanized mice shifted toward smaller compared to that in WT mice (Figure 5D), indicating axonal atrophy in the sciatic nerve of this model. These morphological changes were comparable to those observed in the PMP22-C3 mice (Figure 5A, right panel, E–G).

### 3.5 Changes in plasma NfL levels in humanized mice

**Figure 6:**
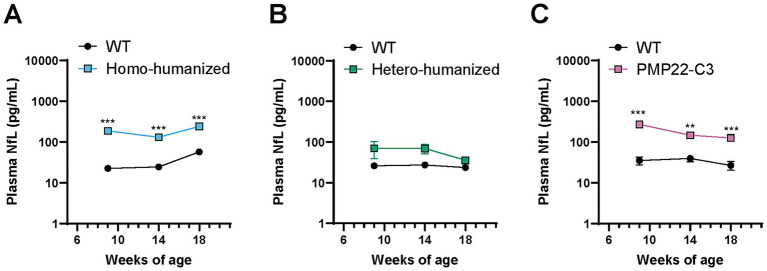
Quantitative analysis of plasma NfL levels in humanized mice and PMP22-C3 mice. Plasma NfL levels were measured using the SiMoA immunoassay in WT, **(A)** homo-humanized, **(B)** hetero-humanized, and **(C)** PMP22-C3 mice at 9, 14, and 18 weeks of age. Data are represented as mean ± SEM (*n* = 8–10/group). Statistical comparisons were performed using two-way Repeated Measures ANOVA with a post hoc Bonferroni’s test (^**^p < 0.01, ^***^p < 0.001 vs. WT mice). WT, wild-type; NfL, neurofilament light chain; SiMoA, single molecule array; SEM, standard error of the mean.

NfL is considered to reflect axonal damage (27), and patients with CMT1A show increased levels of NfL in the blood (28). As axonal atrophy was observed in the sciatic nerves of homo-humanized and PMP22-C3 mice at 18 weeks of age, we investigated plasma NfL levels in these mice at 18 weeks of age and two other earlier points (9 and 14 weeks of age). Individual values of plasma NfL are shown in Supplementary Table S4. Homo-humanized mice showed significantly higher plasma NfL levels than those in WT mice at all ages (8.3-, 5.3-, and 4.2-fold increases at 9, 14, and 18 weeks of age, respectively; *p* < 0.001) (Figure 6A), as well as PMP22-C3 mice (Figure 6C), implying that axonal atrophy occurs earlier than 18 weeks of age. In contrast, hetero-humanized mice showed no statistically significant difference from WT mice (Figure 6B).

### 3.6 Motor disabilities in humanized mice

Motor disabilities such as muscle weakness and impaired balance are common features of patients with CMT1A (29, 30) and mouse models (19, 23, 31, 32). To evaluate whether peripheral nerve dysfunction affects motor ability in the humanized mouse model, we performed three behavioral assessments (grip strength, beam walking, and rotarod tests) at 17 weeks of age.

**Figure 7:**
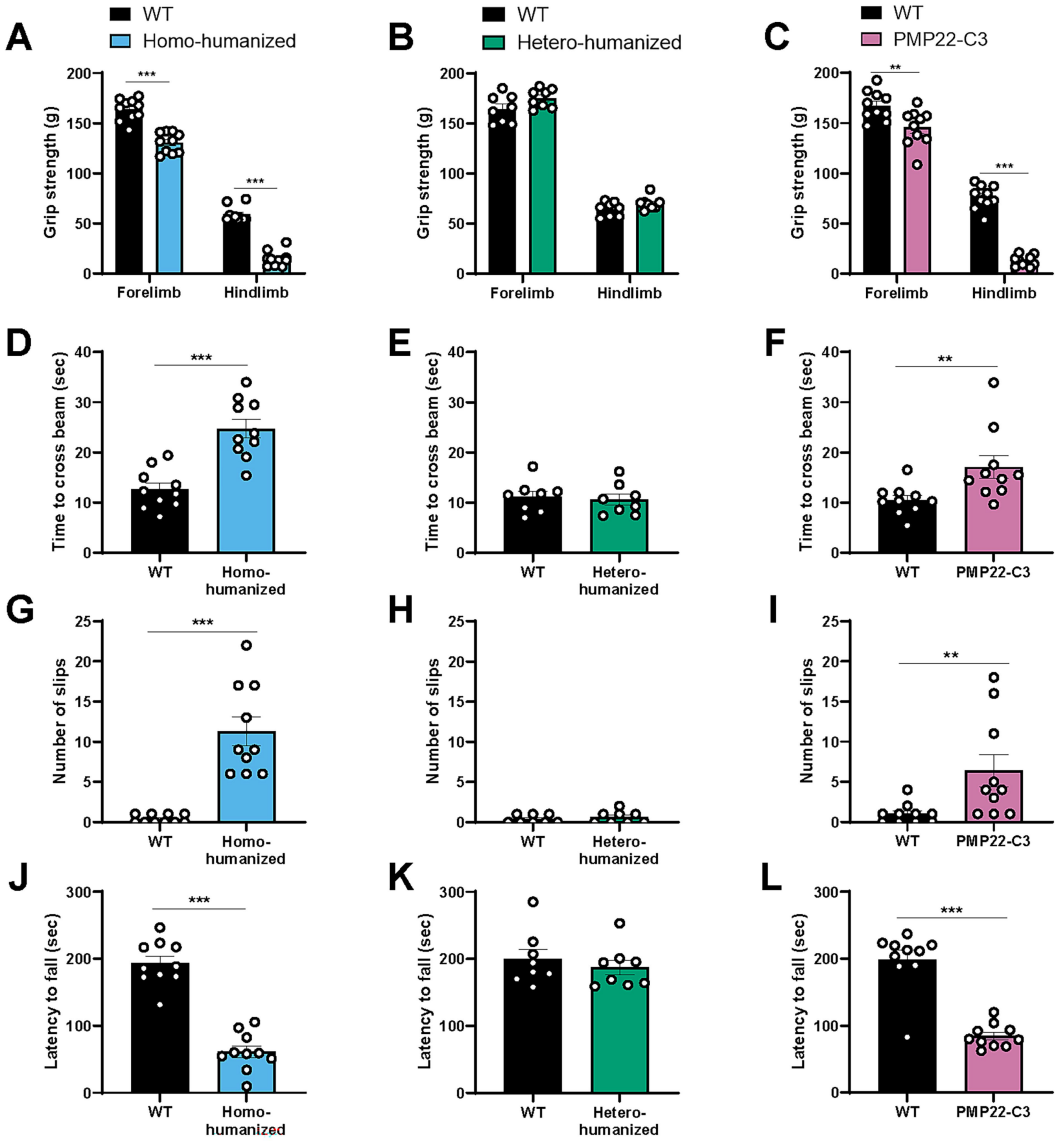
Behavioral characteristics in humanized mice and PMP22-C3 mice. Behavioral assessments were performed at 17 weeks of age. **(A-C)** Grip strength of forelimbs and hindlimbs in WT, **(A)** homo-humanized, **(B)** hetero-humanized, and **(C)** PMP22-C3 mice. Individual data, excluding the maximal and minimal values from five measurements, were averaged. **(D–I)** Time to cross the beam and slip counts of WT, **(D,G)** homo-humanized, **(E,H)** hetero-humanized, and **(F, I)** PMP22-C3 mice in the beam walking test. **(J–L)** Latency to fall off the rotating rod in WT, **(J)** homo-humanized, **(K)** hetero-humanized, and **(L)** PMP22-C3 mice in the rotarod test. Each mouse was tested three times, and the data from Trials 2 and 3 were averaged. Data are represented as mean ± SEM (*n* = 8–10/group). Statistical comparisons were performed using Mann–Whitney U test (^**^p < 0.01, ^***^p < 0.001 vs. WT mice). WT, wild-type; SEM, standard error of the mean.

Homo-humanized mice showed a weaker grip strength in both forelimb (WT: 163.6 ± 3.4 g, homo-humanized: 130.6 ± 3.2 g, *p* < 0.001) and hindlimb (WT: 58.8 ± 2.4 g, homo-humanized: 14.7 ± 2.4 g, *p* < 0.001) than those in WT mice (Figure 7A), while hetero-humanized mice showed a similar level of grip strength as WT mice in both forelimb (WT: 164.3 ± 4.9 g, hetero-humanized: 175.0 ± 3.3 g) and hindlimb (WT: 64.4 ± 2.5 g, hetero-humanized: 70.2 ± 2.3 g) (Figure 7B).

In the beam walking test that assesses the motor balance and gait, WT mice typically crossed the beam in approximately 12 s with less than one slip, while homo-humanized mice took longer to cross the beam (WT: 12.6 ± 1.2 s, homo-humanized: 24.7 ± 1.9 s, *p* < 0.001) (Figure 7D) with many slips (WT: 0.40 ± 0.16, homo-humanized: 11.3 ± 1.8, *p* < 0.001) (Figure 7G), indicating the abnormal motor balance and gait. In contrast, no difference was observed in crossing time (WT: 11.2 ± 1.1 s, hetero-humanized: 10.6 ± 1.1 s) (Figure 7E) or slip counts (WT: 0.38 ± 0.18, hetero-humanized: 0.63 ± 0.26) (Figure 7H) between WT mice and hetero-humanized mice.

The motor coordination was tested using a rotarod. Homo-humanized mice dropped off the rotating rod much earlier than WT mice (WT: 193.3 ± 10.5 s, homo-humanized: 61.1 ± 9.0 s, *p* < 0.001) (Figure 7J). In contrast, hetero-humanized mice showed a latency comparable to that of WT mice (WT: 199.8 ± 14.3 s, hetero-humanized: 187.1 ± 11.2 s) (Figure 7K).

Similar to homo-humanized mice, PMP22-C3 mice showed weaker grip strength in both forelimbs and hindlimbs, longer crossing time and slipping on the beam, and shorter latency to fall off the rod (Figures 7C,F,I,L). These behavioral findings suggested that homo-humanized mice exhibited motor disabilities similar to those of PMP22-C3 mice, whereas hetero-humanized mice maintained normal motor function. This is consistent with the morphological and functional peripheral nerve characteristics observed in all three mouse lines.

### 3.7 Alteration in EIM parameters in the GC muscle of humanized mice

**Figure 8:**
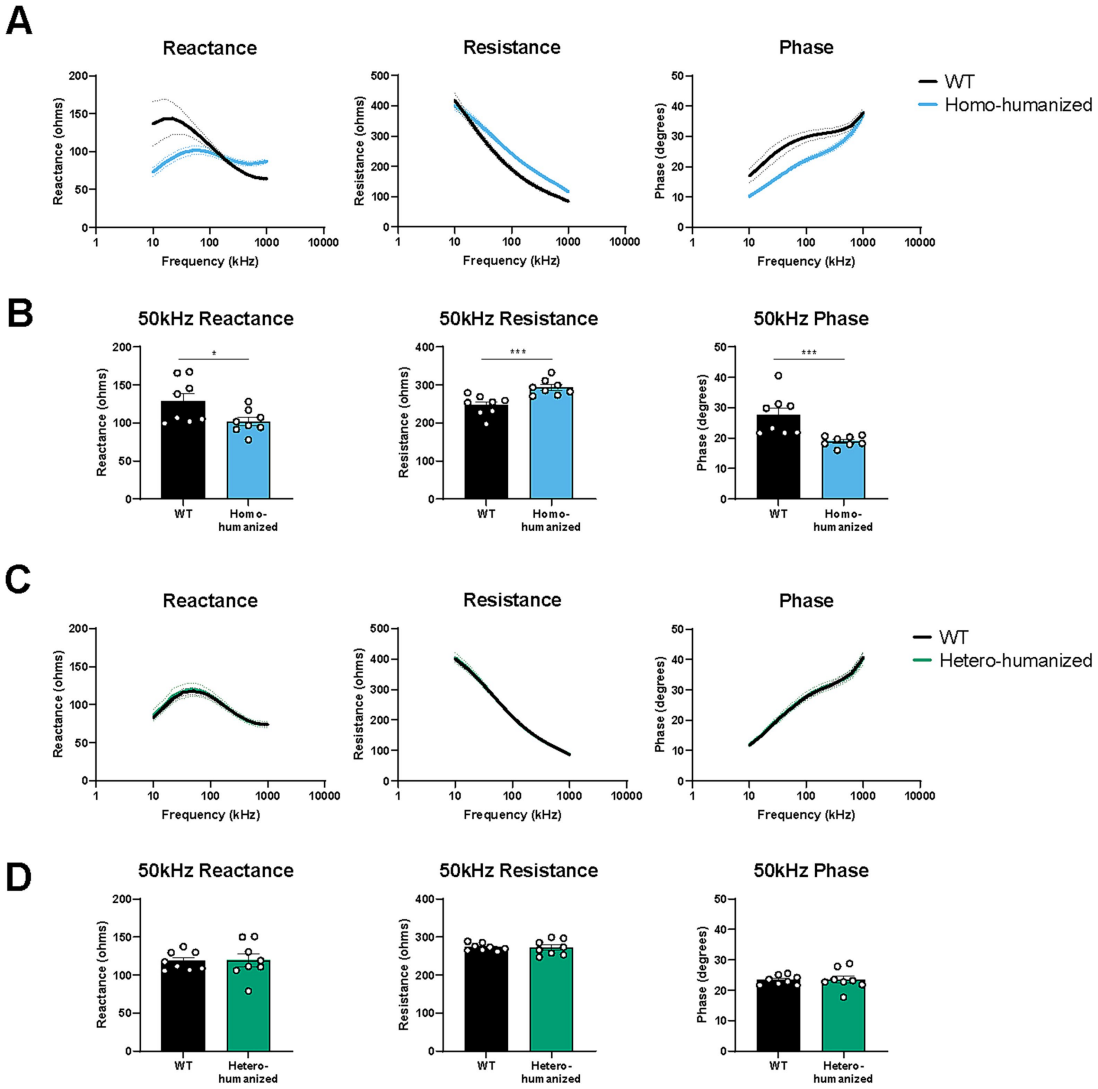
EIM parameters in the GC muscle of humanized mice. Multi-frequency data (10–1,000 kHz) for the EIM parameters were obtained from the GC muscles of WT, **(A)** homo-humanized, and **(C)** hetero-humanized mice at 18 weeks of age. Comparison of EIM parameters at 50 kHz between WT and **(B)** homo-humanized or **(D)** hetero-humanized mice. The EIM data are shown as three parameters: reactance (left), resistance (middle), and phase (right). Data are represented as mean ± SEM (*n* = 8/group). Statistical comparisons were performed using Mann–Whitney U test (^**^p < 0.05, ^**^p < 0.001 vs. WT mice). EIM, electrical impedance myography; WT, wild-type; SEM, standard error of the mean.

We recently reported that PMP22-C3 mice exhibited muscle weakness and alterations in EIM parameters in the GC muscle innervated by tibial nerve branched off from the sciatic nerve (24). To further confirm that muscle weakness can be detected as changes in muscle composition, EIM data were obtained from the GC muscles of WT, homo-humanized, and hetero-humanized mice at 18 weeks of age. Individual values of EIM parameters are shown in Supplementary Table S5. Homo-humanized mice showed obvious alterations in EIM parameters (Figure 8A), which were consistent with EIM changes in PMP22-C3 mice (24). Statistical comparison using EIM parameters at 50 kHz revealed significant decreases in reactance (79.1% of WT mice, *p* < 0.05) and phase (69.0% of WT mice, *p* < 0.001) and an increase in resistance (119.0% of WT mice, *p* < 0.001) in homo-humanized mice than those in WT mice (Figure 8B). In contrast, hetero-humanized mice showed similar values for all parameters as WT mice (Figure 8C), and no statistical difference in the 50 kHz parameters was detected (Figure 8D). As decreased EIM reactance and phase or increased EIM resistance indicate a reduction in muscle fiber size or muscle mass, respectively (33–35), homo-humanized mice have skeletal muscular deterioration, whereas hetero-humanized mice maintain a healthy muscle state.

### 3.8 Morphological changes in the GC muscle of homo-humanized mice

**Figure 9:**
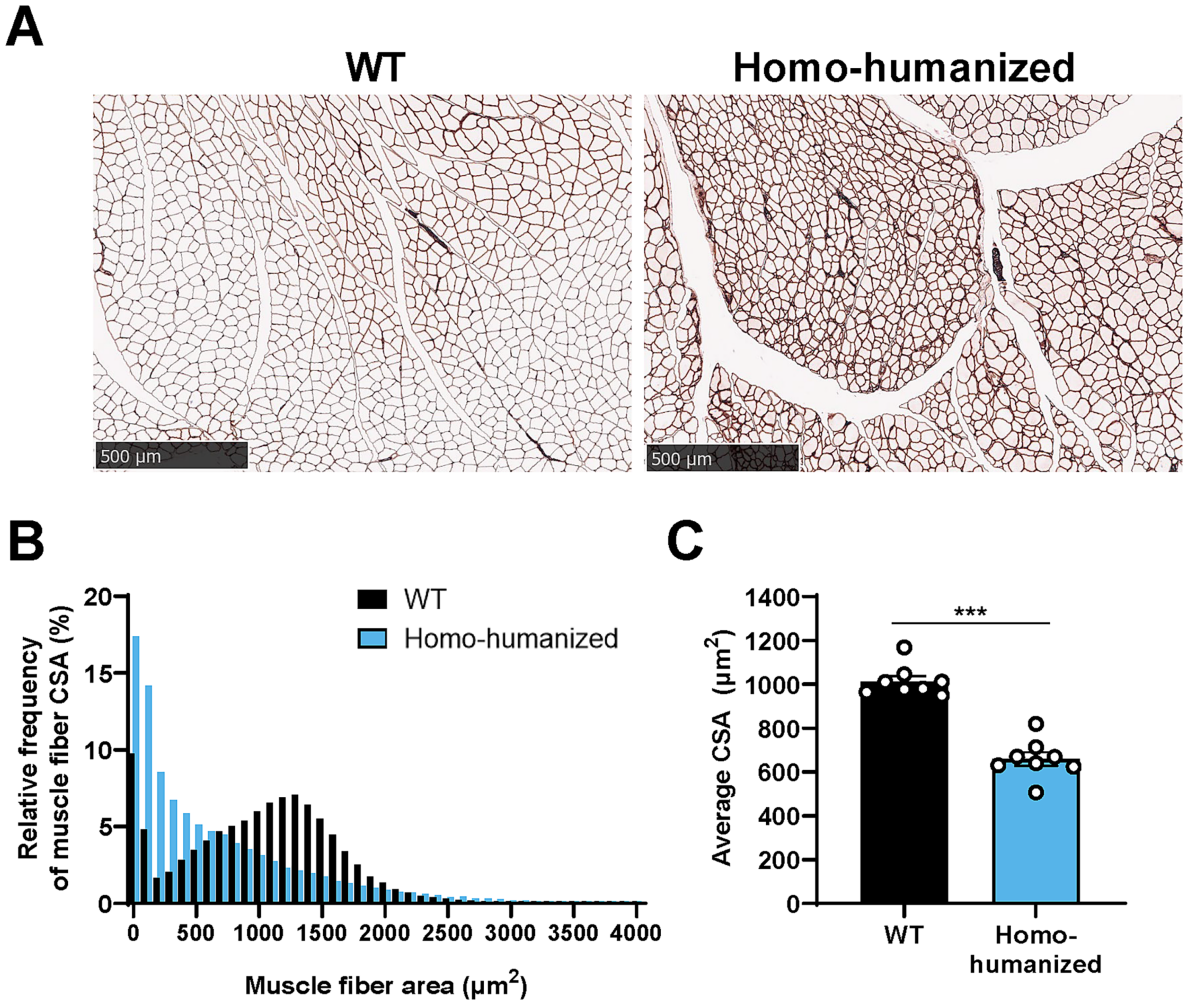
Histopathological analysis of the GC muscle in homo-humanized mice. **(A)** Representative images of laminin-stained cross sections of the GC muscle in WT and homo-humanized mice at 18 weeks of age. **(B)** Comparison of histograms for muscle fiber CSA in the GC muscle of WT and homo-humanized mice. Analyzed muscle fiber frequencies from all animals are plotted for each given muscle fiber CSA with a 100 μm^2^-bin. **(C)** Average muscle fiber CSA in the GC muscle calculated from individual animal values. Data are represented as mean ± SEM (*n* = 8/group). Statistical comparisons were performed using Student’s t-test (^***^p < 0.001 vs. WT mice). GC, gastrocnemius; WT, wild-type; CSA, cross-sectional area; SEM, standard error of the mean.

The histopathology of the GC muscle of homo-humanized mice was investigated at 18 weeks of age to identify morphological changes in muscle fibers underlying EIM alterations. A higher number of thin muscle fibers were observed in homo-humanized mice than in WT mice (Figure 9A). In WT mice, the CSA histogram of individual muscle fibers showed a wider distribution pattern with a peak at approximately 1,300 μm^2^, while homo-humanized mice shifted the distribution to a right-skewed pattern with a peak at the smallest muscle fiber group in the GC muscle (Figure 9B). This distribution change in muscle fibers led to a significant decrease in the average CSA of the GC muscle of homo-humanized mice compared to that in WT mice (65.0% of WT mice, *p* < 0.001) (Figure 9C). These data indicate muscle fiber atrophy in the GC muscles of homo-humanized mice, resulting in alterations in EIM parameters.

## 4 Discussion

In this study, we characterized two humanized PMP22-C3 mouse lines as novel animal models of CMT1A and investigated their utility. Our biochemical, electrophysiological, histopathological, and behavioral studies demonstrated that homo-humanized mice exhibited various aspects of CMT1A pathology.

Quantitative assessments of *PMP22* mRNA and/or PMP22 protein expression levels, compared to the gold-standard PMP22-C3 mouse model, are important for validating the reliability of our newly generated humanized mouse lines. Although PMP22 protein expression can be detected using immunoassays such as western blotting (20), there is room for improvement in its quantitative accuracy. In addition, because PMP22-C3 mice express hPMP22 and mPmp22, the selective detection of each protein is required. In this study, we established a highly sensitive and selective quantitative method using nanoflow liquid chromatography-high-resolution mass spectrometry (nLC-HRMS) for measuring PMP22 protein levels. The chromatogram in Supplementary Figure S1 shows that nLC-HRMS specifically detects hPMP22 and mPmp22. The quantified levels of PMP22 protein correlated with its mRNA levels across mouse models with different genotypes, both hetero- and homo-transgenic, in a gene dosage-dependent manner (Figure 2). Considering that knockdown by *PMP22* mRNA-targeting treatment correlates with phenotypic improvement in CMT1A animal models (19), quantitative measurements are important for estimating the disease state and its change by therapeutic intervention in CMT1A. The mRNA turnover rate is higher than that of protein (36). Therefore, quantifying the PMP22 protein is useful for understanding the time lag between mRNA and protein reduction caused by PMP22-targeting drug candidates. In addition to mRNA detection in the shorter term, the quantitative method for PMP22 protein expression levels will benefit detailed investigations in preclinical models, including dose dependency and time course.

A gene dosage-dependent manifestation of peripheral neuropathy caused by *hPMP22* overexpression was observed in transgenic mouse models of CMT1A (37). The two humanized mouse lines exhibited gene dosage-dependent phenotypes. Homo-humanized mice showed abnormalities in the peripheral nerves and muscles, while hetero-humanized mice, which expressed approximately half the amount of *hPMP22* mRNA/protein compared to homo-humanized mice, did not exhibit any abnormalities. Therefore, the PMP22 levels in hetero-humanized mice were within the normal range. This indicates that the extrinsic hPMP22 protein functions properly at the proper place in mice and that its reduction by 50% or less can normalize the phenotypes of homo-humanized mice. This relationship between PMP22 expression levels and phenotypes would be closer to that of patients with CMT1A (approximately 40% for normalization) than that of PMP22-C3 mice when evaluating drug candidates targeting only hPMP22. Both homo-humanized and PMP22-C3 mice showed similar degrees of peripheral nerve and motor dysfunctions. These findings suggest that intrinsic mPmp22 contributes to the severity of disease phenotypes in PMP22-C3 mice, potentially complicating the efficacy and adverse effects of drug candidates targeting only hPMP22. Regarding the severity of phenotypes, other CMT1A models carrying more *hPMP22* copies, such as C22 mice (7 copies) (22) and TgN248 mice (16 copies) (38), generally have more severe neuropathy phenotypes than mild models including PMP22-C3 mice (3–4 copies), C61 mice (4 copies) (31), and JP18/JY13 mice (2 copies) (39), which may be more relevant to severely affected patients in the CMT1A. In contrast, homo-humanized mice, which exhibit a phenotype comparable to that of PMP22-C3 mice, better mimic the mild myelin and axonal pathology observed in most human patients with CMT1A than a severe model (22). Therefore, with an advantage of overcoming the mPmp22 issue, homo-humanized mice may serve as a more reliable animal model of CMT1A for drug discovery, focusing on a direct approach to hPMP22.

Both homo-humanized and PMP22-C3 mice, but not hetero-humanized mice with lower levels of PMP22 than the homo-genotype, showed alterations in the transcript levels of myelin-related genes, such as *Sc5d*, *Mpz*, and *Pou3f1* in the peripheral nerve, similar to the C22 mouse model (19). This is reasonable because transcriptional changes in C22 mice were ameliorated by treatment with PMP22-targeting ASO (19). The transcriptional changes in myelin-related genes could be associated with the demyelination observed in homo-humanized and PMP22-C3 mice, and smaller myelin areas and increased g-ratio, resulting in slow nerve conduction. In addition, these mouse lines show axonal atrophy, represented by a smaller inner axon area, which could increase plasma NfL levels. The relatively milder increase in plasma NfL compared to other neurodegenerative disease models with robust increases, such as amyotrophic lateral sclerosis (ALS) (40), corresponds to clinical observations that patients with CMT1A show moderate NfL level elevation (28), whereas patients with ALS show a robust increase (41). This could be due to neuropathy confined to the peripheral nerves in PMP22-C3 mice, unlike ALS, which affects both the central and peripheral nervous systems.

Distal muscle weakness/atrophy and balance problems reminiscent of CMT1A (29, 30) were observed in homo-humanized and PMP22-C3 mice but not in hetero-humanized mice (Figure 7). Like PMP22-C3 mice (24), muscular impairments, such as EIM parameter changes and histopathological muscle fiber atrophy, were observed in the hindlimbs of homo-humanized, but not hetero-humanized mice (Figures 8A,B, 9). Considering the selective expression of the extrinsic *hPMP22* gene in peripheral nerves, similar to intrinsic *mPmp22* in PMP22-C3 mice (Supplementary Figure S2), muscle deterioration in homo-humanized mice may stem from peripheral nerve abnormalities caused by *PMP22* overexpression. Since PMP22-C3 mice exhibit progressive muscular deficits similar to those observed in patients (24), the time-dependent changes in muscle deterioration in homo-humanized mice remain to be elucidated in future studies. Collectively, the electrophysiological and histological characterizations indicated that homo-humanized mice mimic the features of neurogenic muscle atrophy and motor disabilities observed in patients with CMT1A.

Given that treatments that excessively reduce PMP22 have the potential to cause HNPP-like symptoms, an accurate estimation of total PMP22 expression is crucial for predicting the therapeutic window of PMP22-targeting drugs between efficacy and adverse effects. As mentioned above, the presence of intrinsic *mPmp22* in existing CMT1A animal models hampers the estimation with drugs targeting hPMP22 but not mPmp22. In contrast, the homo-humanized mice that we developed can simplify the discussion of the relationship between *hPMP22* reduction levels and functional efficacy due to the absence of the *mPmp22* gene. Additionally, since *mPmp22* KO mice, the background mouse line of our humanized mice, show nerve conduction deficits and demyelination, including prominent hypomyelination and tomacula formation (42, 43), homo-humanized mice have the potential to assess the therapeutic effect of a proper reduction of *hPMP22* and the adverse effects of overreduction of *hPMP22*. Further studies investigating the effects of *hPMP22*-targeting drug candidates are required to clarify the utility of this homo-humanized mouse model. Moreover, it will be important to investigate how the duration of *hPMP22* knockdown, as well as the degree of knockdown, impact outcomes in this model.

In conclusion, the newly developed homo-humanized mice exhibited biochemical, electrophysiological, histopathological, and behavioral abnormalities that closely mimicked the features of CMT1A pathology. This model could serve as a promising tool for optimizing the balance between efficacy and potential risks associated with excessive *PMP22* reduction and contributing to the translation of preclinical findings into clinically relevant human treatments and dosing strategies.

## Data Availability

All data generated or analyzed in this study will be made available by the authors, without undue reservation.

## Glossary

ALS: Amyotrophic lateral sclerosis
ASO: Antisense oligonucleotide
CMT1A: Charcot–Marie-Tooth type 1A
CMAP: Compound muscle action potential
CSA: Cross-sectional area
EIM: Electrical impedance myography
GC: Gastrocnemius
hPMP22: Human PMP22
KO: Knockout
nLC-HRMS: Nanoflow liquid chromatography-high-resolution mass spectrometry
mPmp22: Mouse Pmp22
MPZ: Myelin protein zero
NCV: Nerve conduction velocity
NfL: Neurofilament light chain
PMP22: Peripheral myelin protein 22
POU3F1: POU Class 3 Homeobox 1
RT-qPCR: Real-time quantitative polymerase chain reaction
SC5D: Sterol-C5-desaturase
SEM: Standard error of the mean
SiMoA: Single molecule array
WT: Wild-type
