# Supplementary material for: Characterization of a humanized mouse model of Charcot-Marie-Tooth type 1A for the discovery of human PMP22-targeting drugs
Source: Front Neurol. 2025 Oct 29;16:1658204. doi: 10.3389/fneur.2025.1658204 (PMC12606582; doi:10.3389/fneur.2025.1658204)
Supplement: Supplementary file 1 [file Table_1.docx]

**Supplementary Materials**

**Supplementary table**

**Table S1. LC/MS conditions for the analysis of PMP22 protein**

| **LC condition** |  |
| --- | --- |
| **Instruments** | **EASY-nLC 1200 system** |
| **Mobile phase A** | **0.1% formic acid in water** |
| **Mobile phase B** | **0.1% formic acid in acetonitrile/water (8:2, v/v)** |
| **Ratio A/B** | **0 → 30 min; 95/5 → 30/70** |
|  | **30 → 38 min; 30/70 → 50/50** |
|  | **38 → 45 min; 50/50 → 5/95** |
|  | **45 → 55 min; 5/95** |
|  | **55 → 60 min; 5/95 → 95/5** |
| **Flow rate** | **300 nL/min** |
| **Trap column** | **Acclaim PepMap 100 C18 trap column (3 μm, 0.075 mm i.d. × 20 mm, Thermo Fisher Scientific)** |
| **Analytical column** | **NTCC-360/75–3–15 analytical capillary column (3 μm, 0.075 mm i.d. × 150 mm, Nikkyo Technos)** |
| **MS condition** |  |
| **Mode** | **PRM** |
| **Capillary Temp (°C)** | **275** |
| **Spray Voltage** | **Positive Ion (V) = 2,000** |
| **S-Lens RF Level** | **45** |
| **Polarity** | **Positive** |
| **Resolution** | **30,000** |
| **AGC Target** | **2.00E+05** |
| **Maximum IT (ms)** | **54** |
| **Isolation window (m/z)** | **1.6** |
| **Spectrum data type** | **profile** |

**Table S2. MS parameters for the analysis of PMP22 protein**


**Table S3. Individual values of NCV and CMAP in humanized and PMP22-C3 mice**

**Table S4. Individual values of Plasma NfL in humanized and PMP22-C3 mice**


**Table S5. Individual values of EIM parameters in humanized mice**

**Supplementary figures**

**
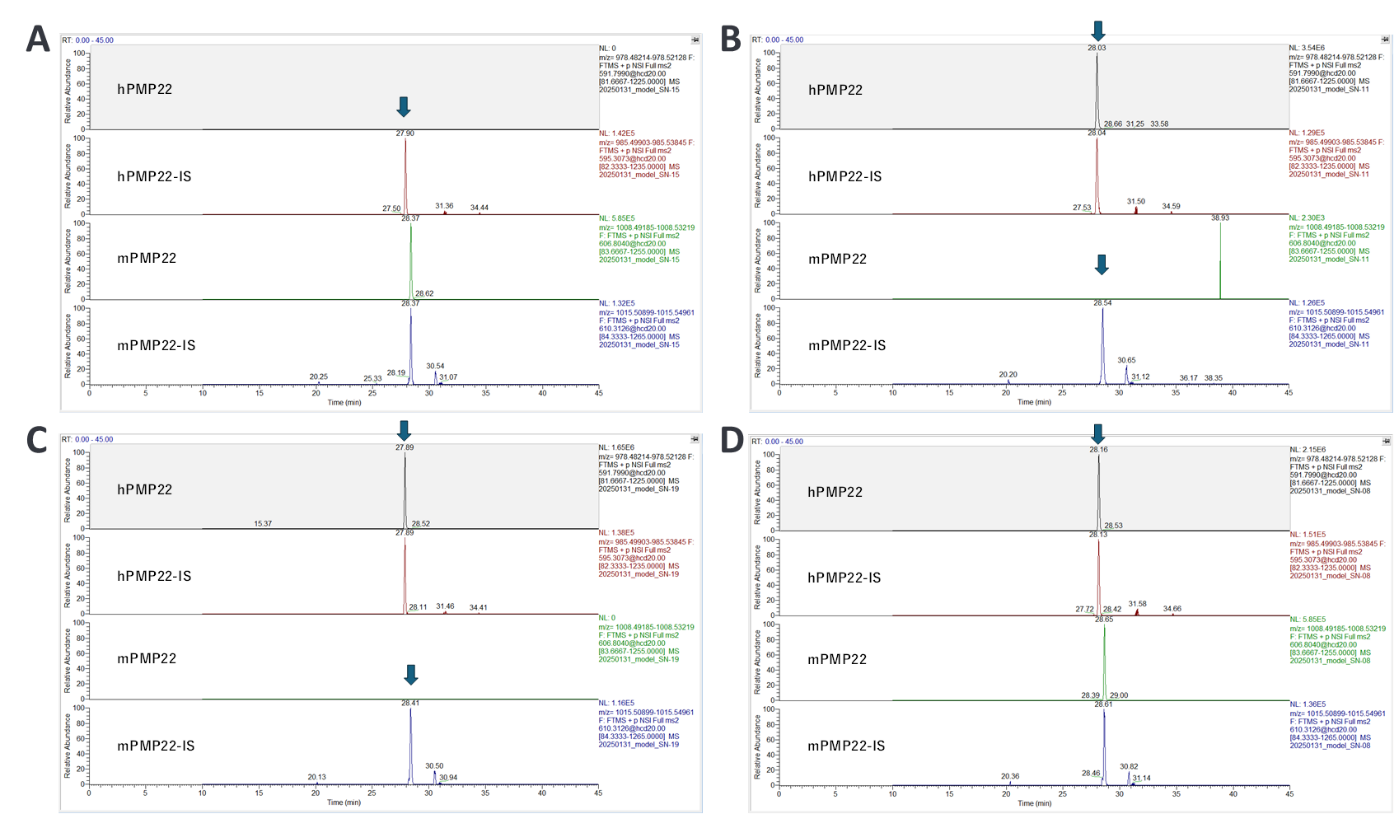
Figure S1**

**Figure S1. Chromatograms of signature peptides for PMP22 protein in sciatic nerve of (A) WT, (B) homo-humanized, (C) hetero-humanized, and (D) PMP22-C3 mice**

**Figure S2**

**Figure S2. Expression of *hPMP22/mPmp22* mRNA in the sciatic nerve and the GC muscle of PMP22-C3 mice**
